# Physiological and Transcriptome Analyses Reveal the Important Role of Microbial Fertilizer in the Response of Sugar Beet Seedlings to Saline-Alkali Stress

**DOI:** 10.3390/ijms26188840

**Published:** 2025-09-11

**Authors:** Chunyan Huang, Kang Han, Xiaoxia Guo, Lu Tian, Caiyuan Jian, Wenbin Su, Zhigang Wei, Peng Zhang, Yinghao Li, Huimin Ren, Jianjun Song, Liang Wang, Yongkang Zhang, Zhi Li

**Affiliations:** 1 Institute of Speciality Crops, Inner Mongolia Academy of Agricultural and Animal Husbandry Sciences, Zhao Jun Road Num. 22, Yu Quan District, Hohhot 010031, China; hcy86@aliyun.com (C.H.);; 2 Institute of Speciality Crops, Bayannur Academy of Agricultural & Animal Sciences, Linshan Road Kilometer 9, Linhe District, Bayannur 015400, China; 3 Institute of Economic Crops, Agriculture and Forestry Sciences of Ulanqab, Pingdiquan Town, Chayuqian Banner, Jining 012000, China

**Keywords:** sugar beet, physiological analysis, transcriptome analysis, microbial fertilizer, saline–alkali stress

## Abstract

Sugar beet is one of China’s major cash crops, and Inner Mongolia has become an important sugar base in China. However, cultivation of sugar beet in Inner Mongolia could be improved, as it contains 1.06 million hectares of saline–alkali land, accounting for 11.4% of the total arable land in the region. This saline–alkali land challenges the potential for sugar-beet cultivation as excessive concentrations of saline and alkaline substances, in addition to ionic components, have been demonstrated to have a detrimental effect on the growth of crops, including sugar beet. In sugar beet, excessive concentrations of saline and alkaline substances impact the normal metabolism of sugar beets, thereby inhibiting their growth and development. The present study posits that the utilization of a microbial fertilizer has the potential to mitigate the repercussions of saline–alkali stress. The application of microbial fertilizer has been demonstrated to exert a substantial influence on the accumulation of soluble sugars, soluble proteins and free proline in sugar beet roots and leaves. This study demonstrated a decrease in malondialdehyde (MDA) content and an increase in the K^+^/Na^+^ ratio following treatment with a microbial fertilizer. Furthermore, increased activity of superoxide dismutase (SOD), peroxidase (POD) and catalase (CAT) enzymes was observed. These changes induced an increase in the contents of indole-3-acetic acid (IAA), gibberellic acid (GA) and zeatin (ZR) and a decrease in abscisic acid (ABA) content. The results also indicate an increase in the seedling retention rate and fresh weight of sugar beets.

## 1. Introduction

The area of salinized cropland in Inner Mongolia has been estimated to be 1.06 million hectares, accounting for 11.4% of the total cropland area in the region. Soil salinization has been shown to induce physiological drought in plants, thereby impacting their capacity to absorb nutrients and water. The presence of excessive saline and alkaline components in the soil has been demonstrated to adversely affect plant growth [1,2]. Furthermore, the stress caused by alkaline salts has been found to result in significantly more pronounced growth inhibition compared to neutral salt stress [3]. This inhibition primarily manifests in the suppression of the growth and differentiation of plant tissues and organs [4] and the progression of plant developmental processes. The impact on leaf morphology is particularly evident [5]. The root system is the plant organ that is directly injured by saline–alkali conditions [6], and it is more sensitive to the soil environment [7]. Furthermore, elevated soil Na^+^ content has been demonstrated to diminish the water absorption capacity of plants [8,9], engendering nutrient deficiencies [10], growth and development inhibition and, consequently, a substantial reduction in crop yield. This, in turn, exerts a significant impact on agricultural development, thereby influencing the evolution of the social economy.

The low osmotic potential of saline–alkali soils produces osmotic stress in plants. This stress is overcome by the synthesis of regulatory substances [11], including soluble sugars, proline and betaine. The accumulation of these substances is rapid and ensures that photosynthesis can proceed normally [12,13]. As a product of photosynthesis, soluble sugars act primarily as the vital energy metabolites of organisms; however, these sugars are also significant in the osmoregulation of plants under adverse conditions. Enzymatic defense systems in plants have been shown to resist the large accumulation of reactive oxygen species in the body to avoid plant injury [14,15,16]. Such systems include SOD, POD and CAT enzymes, which interact with each other to maintain reactive oxygen species levels in the organism within a certain range. This, in turn, protects the plant or reduces the damage from saline–alkali stress. In addition to regulation of ions, plant hormones also play a role in salt tolerance, and the relationship between salt tolerance and hormone content in plants is a subject of considerable interest. The role of hormones, such as growth hormone and gibberellin, in promoting growth is well documented, while the function of abscisic acid, often referred to as the ‘adversity hormone’, is less understood. However, it is evident that plants possess the ability to modify the levels of various hormones within their bodies to ensure normal growth and development [17]. In the presence of adversity stress, plants initially respond at the gene level by mobilizing a significant number of genes to become co-woven into a complex regulatory network. This process results in the production of corresponding proteins, which, in turn, regulate metabolite synthesis and maintain the mechanistic balance of the plant body [18].

The field of bioremediation has become a prominent area of interest in contemporary scientific discourse, with the development of microbial processes for the improvement of saline and alkaline land being a particularly salient research direction. The utilization of microbial fertilizer, which is produced by microorganisms through the fermentation process, has been demonstrated to contain a substantial quantity of active beneficial microorganisms. These microorganisms have the capacity to supply nutrients to the plant body and regulate plant growth through their specific role in the ecosystem. The presence of beneficial microorganisms in microbial fertilizer has been shown to facilitate the conversion of substances that are not otherwise absorbable by plants into nutrients that can be absorbed and utilized by them. This process can result in a fertilizing effect, thereby serving as a beneficial supplement to chemical fertilizer. Furthermore, the metabolic processes of beneficial microorganisms have been observed to yield plant-stimulating substances, which is an inherent property of these microorganisms [19]. The utilization of microbial fertilizer has been demonstrated to facilitate the release of insoluble mineral nutrients, thereby enhancing the absorption of nutrients by plants. Furthermore, microbial fertilizer has been shown to contribute to the improvement of soil fertility, the enhancement of plant resilience and the augmentation of crop quality and yields [20,21].

Sugar beet (*Beta vulgaris*) is a biennial herbaceous plant from the genus Beta and family Marantaceae. It is a unique cash crop in the north of China, exhibiting drought, cold and saline–alkali tolerance. It is a widely adapted crop with strong resistance and high economic value. The saline–alkali tolerance of sugar beet is considered to be among the most advanced of all cultivated crops. Indeed, it is the crop of choice for the development and utilization of saline and alkaline land, including salinized cropland in Inner Mongolia [22]. Previous studies on sugar beet have focused on its tolerance and physiological responses to saline and alkaline stress and the regulatory effects of exogenous substances on saline–alkali stress [23,24,25]. However, there have been very few studies on the effects of microbial fertilizer on the physiological characteristics and transcriptomics of sugar beet under saline–alkali stress. This paper has several objectives, as follows: to study the mechanism of the protective effect of compound microbial fertilizer on the physiological properties and transcriptomics of saline–alkali-stressed sugar beet; to elucidate the regulatory mechanism of this fertilizer at the physiological and molecular levels; to clarify the metabolic pathway and enhance the mechanisms of the microbial fertilizer in regulating saline–alkali-stressed sugar beet; and to provide a theoretical basis for the cultivation of sugar beet under saline–alkali conditions.

## 2. Results

### 2.1. Impact of Microbial Fertilizer on the Growth of Saline–Alkali-Stressed Sugar Beet Seedlings

**Figure 1:**
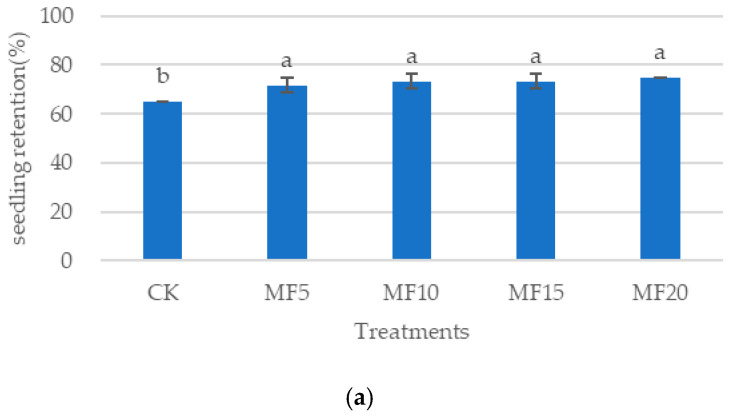

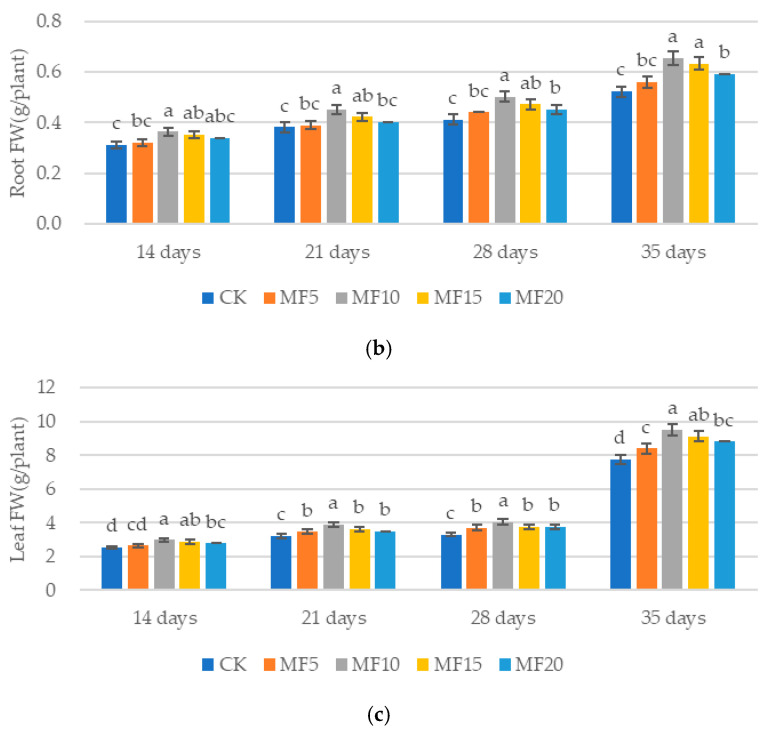
The impact of microbial fertilizer on seedling retention (**a**), root fresh weight (FW) (**b**) and leaf FW (**c**) of saline–alkali-stressed sugar beet seedlings. CK, MF5, MF10, MF15 and MF20 represent 0 kg, 5 kg, 10 kg, 15 kg and 20 kg microbial fertilizer applied to 300 kg saline–alkali soil, respectively; 14 days, 21 days, 28 days and 35 days correspond to the number of days after seedling emergence. Data are presented as the mean ± SD value, and lowercase letters represent significant differences among the means of the microbial fertilizer treatments (*p* ≤ 0.05).

As microbial fertilizer application increased, a gradual upward trend was observed in the sugar beet seedling retention rate across all five treatments. Compared with the CK, the sugar beet seedling retention rate exhibited a significant increase with all microbial fertilizer treatments; however, the difference among the different microbial fertilizer treatments was not significant (Figure 1a). As microbial fertilizer application increased, both the root fresh weight and leaf fresh weight of the sugar beets exhibited an upward trend, followed by a subsequent decrease (Figure 1b,c). Among the various treatments, MF10 demonstrated the most significant outcomes, with the root fresh weight at 14 days, 21 days, 28 days and 35 days increasing by 15.63%, 18.40%, 22.03% and 24.98%, respectively, relative to the CK. Concurrently, the leaf fresh weight for the MF10 treatment increased by 18.12%, 21.76%, 22.44% and 22.66%, respectively, relative to the CK. This finding suggests that the microbial fertilizer may have the capacity to alleviate the inhibition of saline–alkali stress on the growth of sugar beet seedlings and promote the growth of sugar beet plants.

### 2.2. Impact of Microbial Fertilizer on Osmoregulatory Substances in Saline–Alkali-Stressed Sugar Beet Seedlings

**Figure 2:**
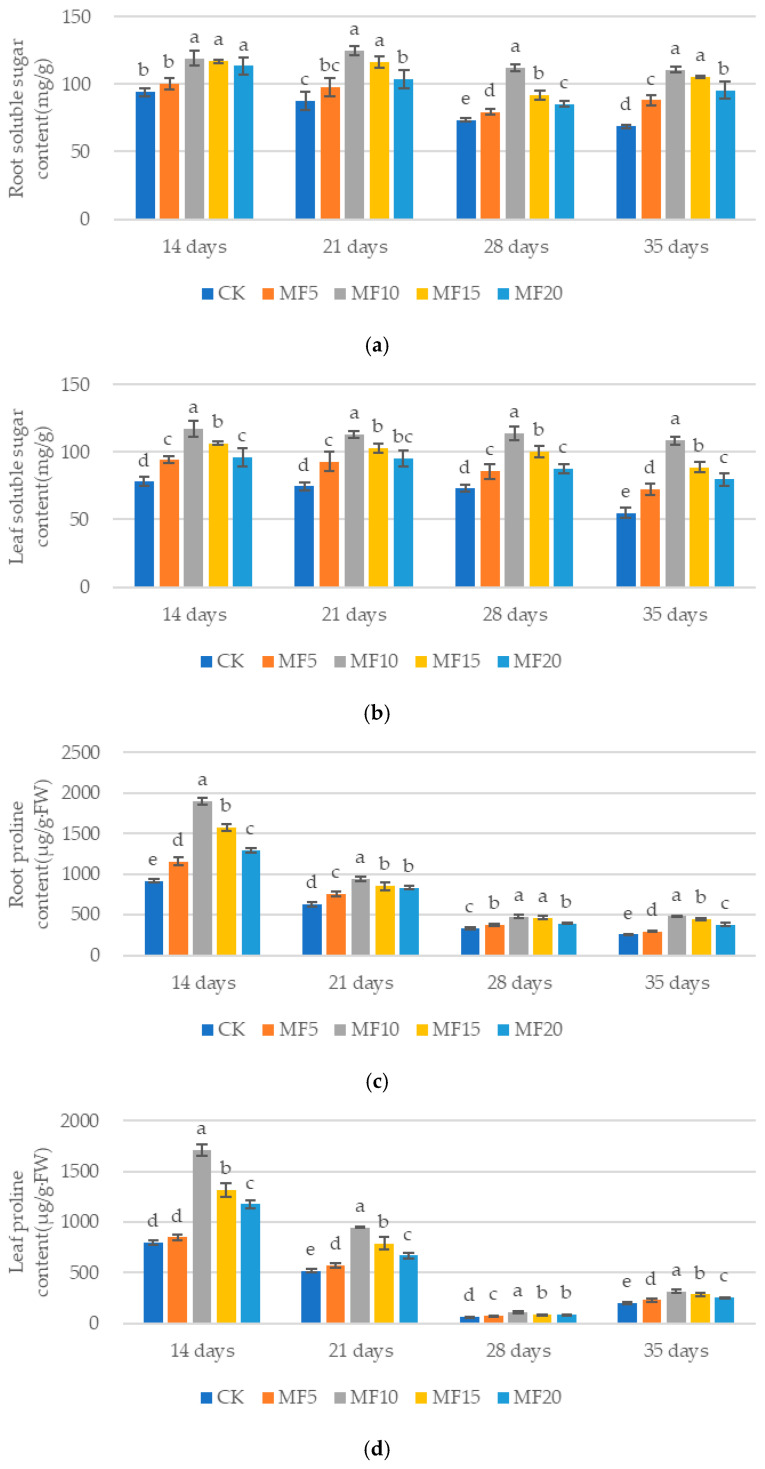
The impact of microbial fertilizer on the root soluble sugar (**a**), leaf soluble sugar (**b**), root proline (**c**) and leaf proline (**d**) contents of saline–alkali-stressed sugar beet seedlings. CK, MF5, MF10, MF15 and MF20 represent 0 kg, 5 kg, 10 kg, 15 kg and 20 kg microbial fertilizer applied to 300 kg saline–alkali soil, respectively; 14 days, 21 days, 28 days and 35 days correspond to the number of days after seedling emergence. Data are presented as the mean ± SD value, and lowercase letters represent significant differences among the means of the microbial fertilizer treatments (*p* ≤ 0.05).

As microbial fertilizer application increased, the soluble sugar and free proline contents of the sugar beets exhibited an initial upward trend across the five treatments, followed by a subsequent decline (Figure 2a–d). Among these treatments, MF10 demonstrated the most significant increases. Compared to the CK, the soluble sugar content of sugar beet roots increased by 26.39%, 42.39%, 47.86% and 60.92% on days 14, 21, 28 and 35, respectively. Similarly, the soluble sugar content of sugar beet leaves increased by 50.30%, 51.61%, 52.37% and 98.03%, respectively, relative to the CK. The free proline content of the sugar beet root system increased by 107.84%, 50.85%, 41.15% and 87.85%, respectively, and that of the leaves by 115.31%, 82.79%, 77.18% and 58.55%, respectively, compared with the CK. These findings suggest that microbial fertilizer can stimulate the synthesis of soluble sugars and free proline in sugar beets.

**Figure 3:**
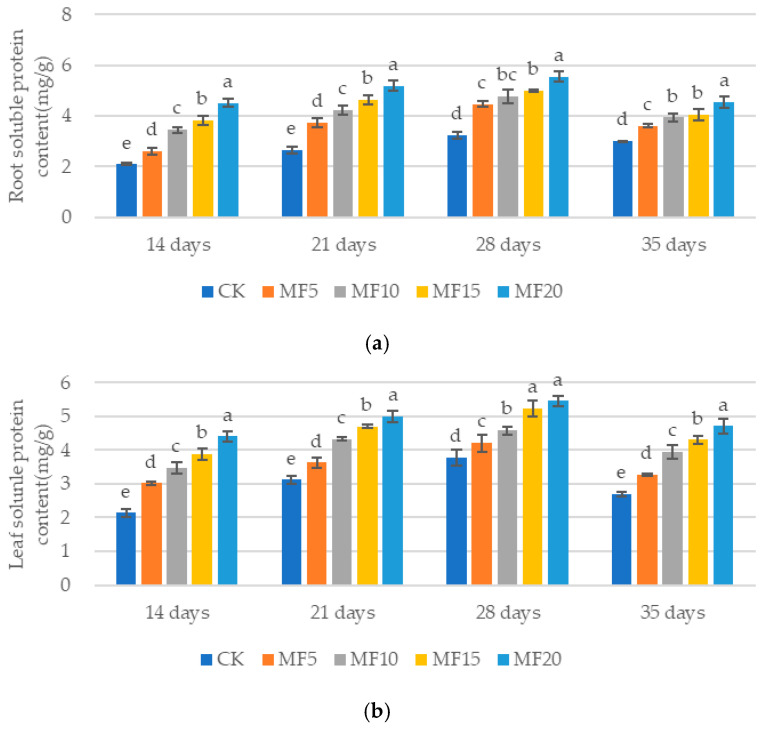
The impact of microbial fertilizer on the root (**a**) and leaf (**b**) soluble protein contents of saline–alkali-stressed sugar beet seedlings. CK, MF5, MF10, MF15, and MF20 represent 0 kg, 5 kg, 10 kg, 15 kg and 20 kg microbial fertilizer applied to 300 kg saline–alkali soil, respectively; 14 days, 21 days, 28 days and 35 days correspond to the number of days after seedling emergence. Data are presented as the mean ± SD value, and lowercase letters represent significant differences among the means of the microbial fertilizer treatments (*p* ≤ 0.05).

The soluble protein content of the sugar beets exhibited a gradual increase with the increase in microbial fertilizer application (Figure 3a,b). Compared with the CK, the soluble protein contents of sugar beet roots and leaves were found to be significantly increased in each microbial fertilizer treatment, with the increases ranging from 24.28 to 116.41%, 39.68 to 94.88%, 37.23 to 70.62% and 20.69 to 51.51% at 14 days, 21 days, 28 days and 35 days, respectively. Correspondingly, the values obtained for the leaf system increased from 41.18 to 105.94%, 16.23 to 60.20%, 11.29 to 43.55% and 21.14 to 74.98%, respectively, relative to the CK. These findings suggest that microbial fertilizer may be a beneficial addition in the cultivation of sugar beets, potentially enhancing the soluble protein content in the plant.

**Figure 4:**
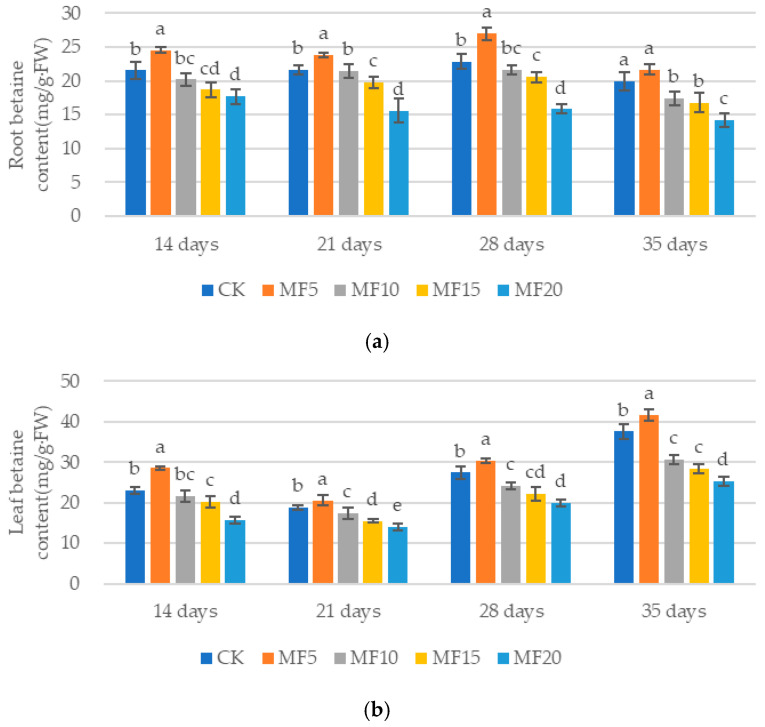
The impact of microbial fertilizer on the root (**a**) and leaf (**b**) betaine contents of saline–alkali-stressed sugar beet seedlings. CK, MF5, MF10, MF15 and MF20 represent 0 kg, 5 kg, 10 kg, 15 kg and 20 kg microbial fertilizer applied to 300 kg saline–alkali soil, respectively; 14 days, 21 days, 28 days and 35 days correspond to the number of days after seedling emergence. Data are presented as the mean ± SD value, and lowercase letters represent significant differences among the means of the microbial fertilizer treatments (*p* ≤ 0.05).

With the increase in microbial fertilizer application, the order of the five treatments in terms of sugar beet root and leaf betaine contents was MF5 > CK > MF10 > MF15 > MF20 (Figure 4a,b). In the MF5 treatment, the betaine content of the beet root system increased by 13.99%, 9.90%, 18.15% and 8.52%, respectively, and that of the leaves increased by 23.40%, 8.69%, 10.57% and 10.55%, respectively, compared to the CK. These findings suggest that microbial fertilizer can enhance the accumulation of betaine in the cytoplasm by increasing the activity of betaine aldehyde dehydrogenase, thereby improving the adaptability to saline–alkali stress. However, as microbial fertilizer application increased, the accumulation of betaine was found to be inhibited.

**Figure 5:**
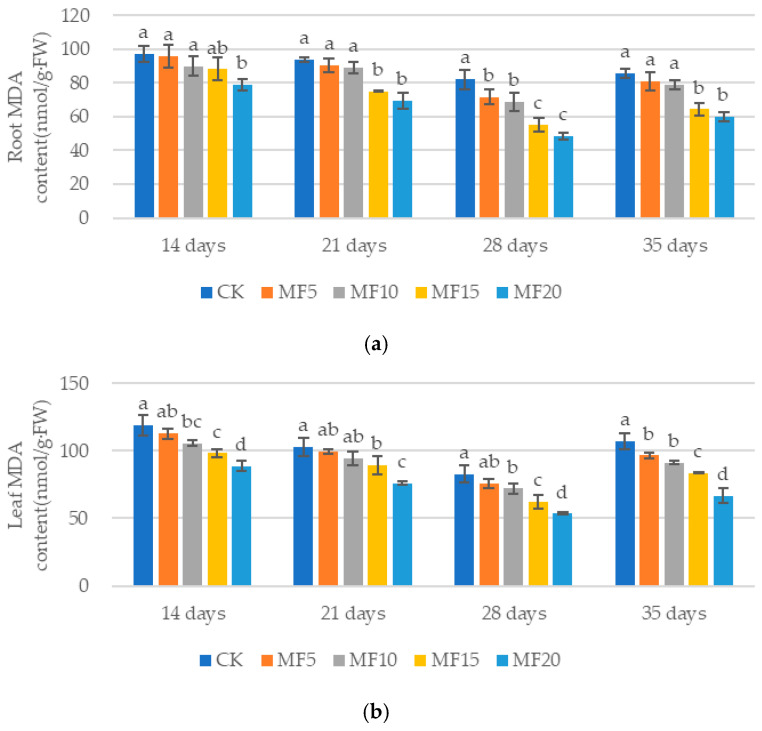
The impact of microbial fertilizer on the root (**a**) and leaf (**b**) MDA contents of saline–alkali-stressed sugar beet seedlings. CK, MF5, MF10, MF15 and MF20 represent 0 kg, 5 kg, 10 kg, 15 kg and 20 kg microbial fertilizer applied to 300 kg saline–alkali soil, respectively; 14 days, 21 days, 28 days and 35 days correspond to the number of days after seedling emergence. Data are presented as the mean ± SD value, and lowercase letters represent significant differences among the means of the microbial fertilizer treatments (*p* ≤ 0.05).

MDA is a product of membrane peroxidation that has been shown to directly reflect the degree of membrane damage. As microbial fertilizer application increased, gradual decreases in the MDA contents of the sugar beet roots and leaves were observed in all four fertilizer treatments compared to the CK (Figure 5a,b). The root percentage of MDA decreased by 1.58–18.94%, 3.60–25.73%, 12.51–40.75% and 5.50–29.86% at 14 days, 21 days, 28 days and 35 days, respectively. Additionally, the leaf percentage decreased by 5.26–25.25%, 3.49–26.19%, 8.26–35.44% and 9.94–37.59%, respectively, relative to the CK. The findings of this study indicate that microbial fertilizer is beneficial for reducing the damage caused by saline–alkali stress on the membrane system of sugar beets.

**Figure 6:**
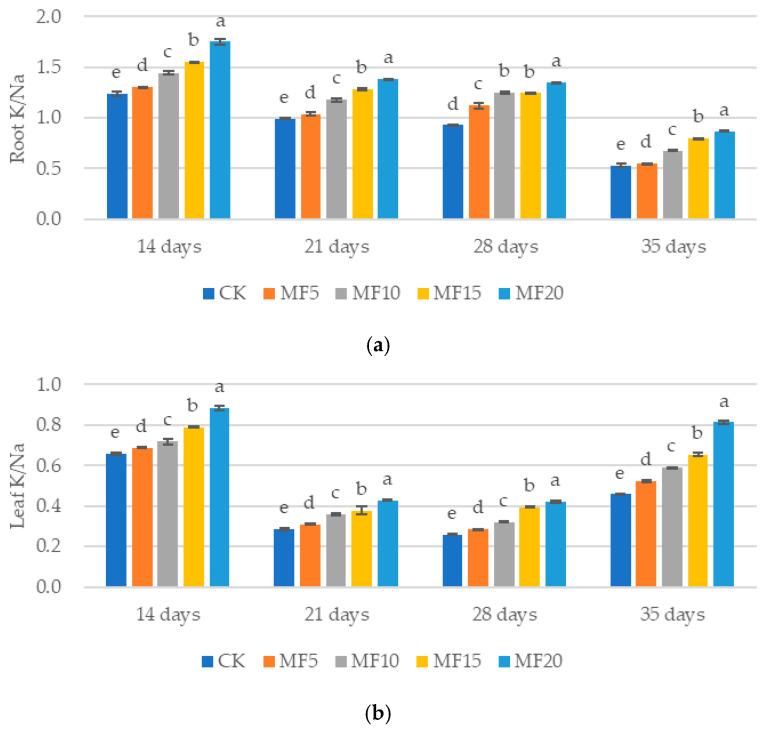
The impact of microbial fertilizer on the root (**a**) and leaf (**b**) K/Na ratio of saline–alkali-stressed sugar beet seedlings. CK, MF5, MF10, MF15 and MF20 represent 0 kg, 5 kg, 10 kg, 15 kg and 20 kg microbial fertilizer applied to 300 kg saline–alkali soil, respectively; 14 days, 21 days, 28 days and 35 days correspond to the number of days after seedling emergence. Data are presented as the mean ± SD value, and lowercase letters represent significant differences among the means of the microbial fertilizer treatments (*p* ≤ 0.05).

As microbial fertilizer application increased, a gradual upward trend in the K^+^/Na^+^ ratio was observed in the sugar beets across all five treatments (Figure 6a,b). For instance, the K^+^/Na^+^ ratios in the sugar beet roots were recorded as 1.24, 1.30, 1.44, 1.55 and 1.75 at 14 days after seedling emergence for the CK, MF5, MF10, MF15 and MF20, respectively. Similarly, the K^+^/Na^+^ ratios in the sugar beet leaves were 0.66, 0.69, 0.72, 0.79 and 0.88, respectively. These data indicate a general upward trend in root K^+^/Na^+^ ratio relative to those in the leaves. This finding suggests that microbial fertilizer may enhance the K^+^/Na^+^ ratio in sugar beets, thereby contributing to the maintenance of an optimal ionic balance within the plant.

### 2.3. Impact of Microbial Fertilizer on Antioxidant Enzyme Activity in Saline–Alkali-Stressed Sugar Beet Seedlings

**Figure 7:**
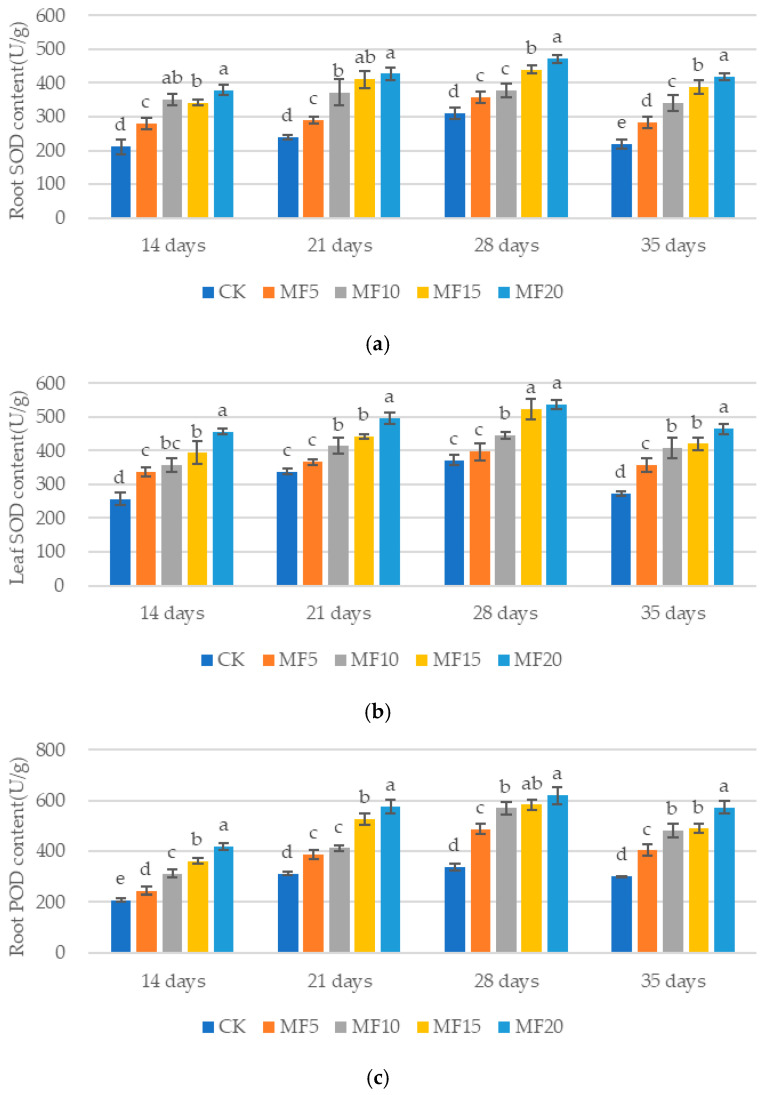

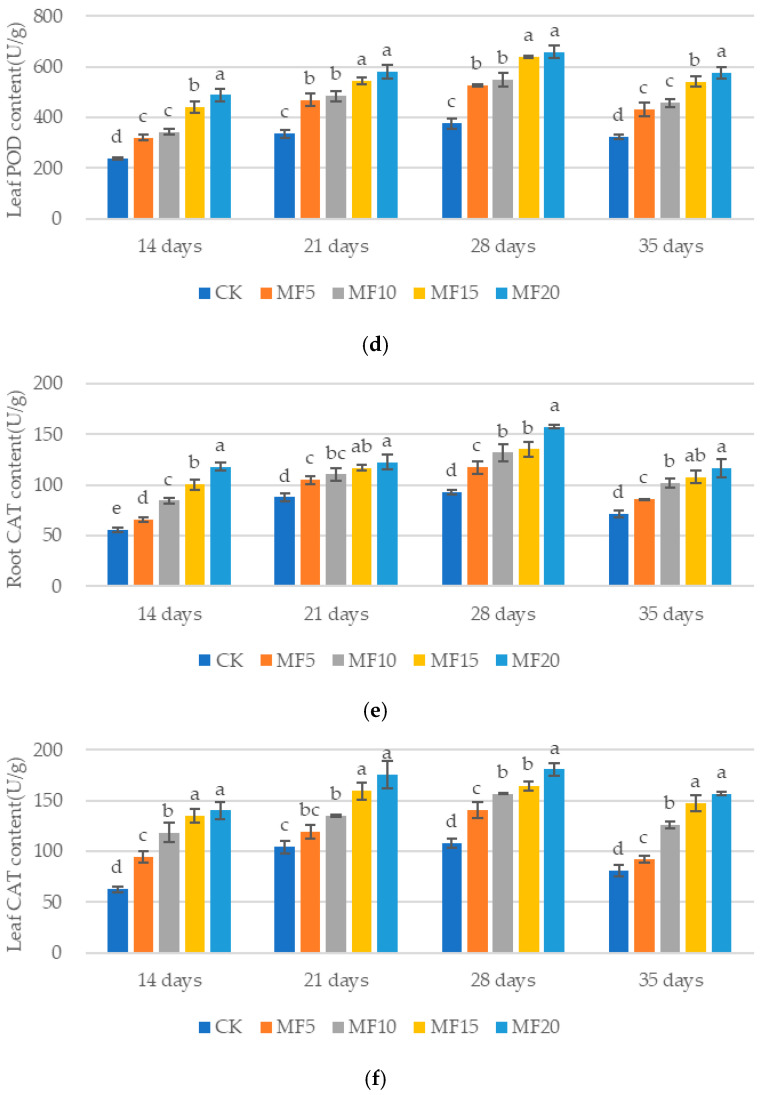
The impact of microbial fertilizer on the soluble enzyme content of saline–alkali-stressed sugar beet seedlings: root SOD (**a**); leaf SOD (**b**); root POD (**c**); leaf POD (**d**); root CAT (**e**); leaf CAT (**f**). CK, MF5, MF10, MF15 and MF20 represent 0 kg, 5 kg, 10 kg, 15 kg and 20 kg microbial fertilizer applied to 300 kg saline–alkali soil, respectively; 14 days, 21 days, 28 days and 35 days correspond to the number of days after seedling emergence. Data are presented as the mean ± SD value, and lowercase letters represent significant differences among the means of the microbial fertilizer treatments (*p* ≤ 0.05).

The application of microbial fertilizer resulted in a gradual increase in the SOD, POD and CAT levels of sugar beets across all five treatments. Compared with the CK, the SOD activities of sugar beet roots and leaves were found to be significantly elevated in each microbial fertilizer treatment (except for MF5 leaves at 14 d and 28 d) (Figure 7a,b). The increases observed in the roots ranged from 32.64 to 79.83%, 21.02 to 78.74%, 14.51 to 51.34% and 29.88 to 91.00% for 14 days, 21 days, 28 days and 35 days, respectively, relative to the CK. The increases observed in the leaves ranged from 31.39 to 77.30%, 8.48 to 46.45%, 6.55 to 44.11% and 30.57 to 70.16%, respectively, relative to the CK. This study demonstrated that both root and leaf POD levels increased significantly in sugar beet (Figure 7c,d), with the increases in roots ranging from 18.17 to 101.97%, 23.41 to 84.51%, 43.68 to 83.05% and 34.22 to 91.04% and those in leaves ranging from 32.43 to 104.28%, 39.81 to 72.86%, 40.02 to 74.99% and 33.94 to 78.26%, respectively, for 14 days, 21 days, 28 days and 35 days, relative to the CK. The present study demonstrated that the root and leaf CAT levels of sugar beet were significantly increased (except for MF5 leaves at 14 d) by 18.00–111.18%, 19.62–39.57%, 26.91–70.33% and 19.72–63.09% for the roots and 51.09–124.28%, 14.23–68.13%, 30.50–67.76% and 14.08–94.44% for the leaves, respectively, for 14 days, 21 days, 28 days and 35 days, relative to the CK (Figure 7e,f). These findings suggests that microbial fertilizer can significantly increase the antioxidant enzyme levels of sugar beet, thereby enhancing its tolerance to adverse stress.

### 2.4. Impact of Microbial Fertilizer on Hormone Content in Saline–Alkali-Stressed Sugar BEET Seedlings

**Figure 8:**
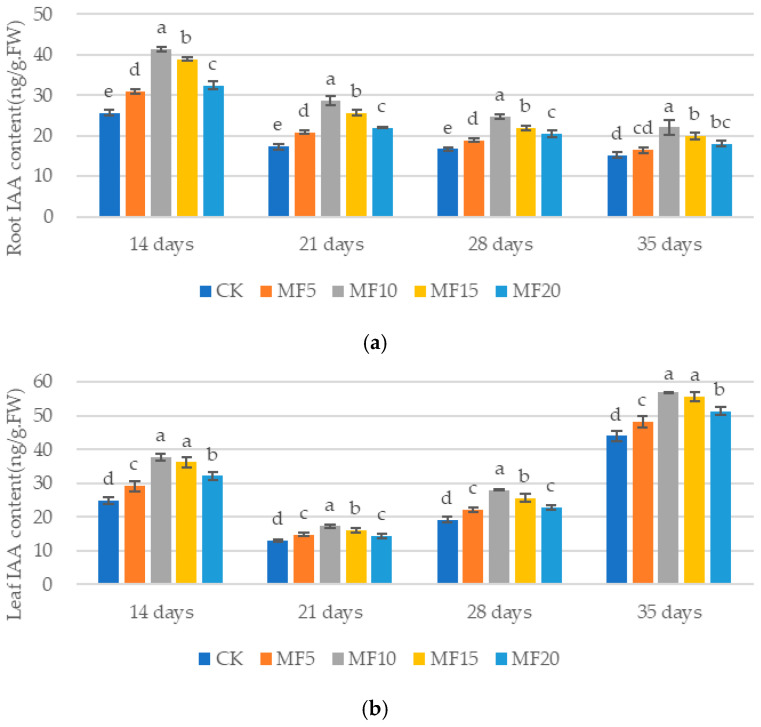
The impact of microbial fertilizer on the root (**a**) and leaf (**b**) IAA contents of saline–alkali-stressed sugar beet seedlings. CK, MF5, MF10, MF15 and MF20 represent 0 kg, 5 kg, 10 kg, 15 kg and 20 kg microbial fertilizer applied to 300 kg saline–alkali soil, respectively; 14 days, 21 days, 28 days and 35 days correspond to the number of days after seedling emergence. Data are presented as the mean ± SD value, and lowercase letters represent significant differences among the means of the microbial fertilizer treatments (*p* ≤ 0.05).

Endogenous hormones are signaling substances with complex and diverse physiological mechanisms. Of these, IAA has been demonstrated to promote plant growth and development. As microbial fertilizer application increased, a trend of increasing and then decreasing IAA content was observed across the five treatments. The IAA content of each microbial fertilizer treatment was significantly higher than that of the CK (except for the MF5 roots at 35 days) (Figure 8a,b). MF10 was the most effective; compared to the CK, the IAA content of MF10 sugar beet roots increased by 61. 51%, 65.33%, 47.64% and 45.00% at 14 days, 21 days, 28 days and 35 days, respectively. Meanwhile, the IAA content of MF10 sugar beet leaves increased by 51.78, 32.98%, 46.33% and 29.32%, respectively. These findings suggest that microbial fertilizer can stimulate the synthesis of IAA in sugar beets.

**Figure 9:**
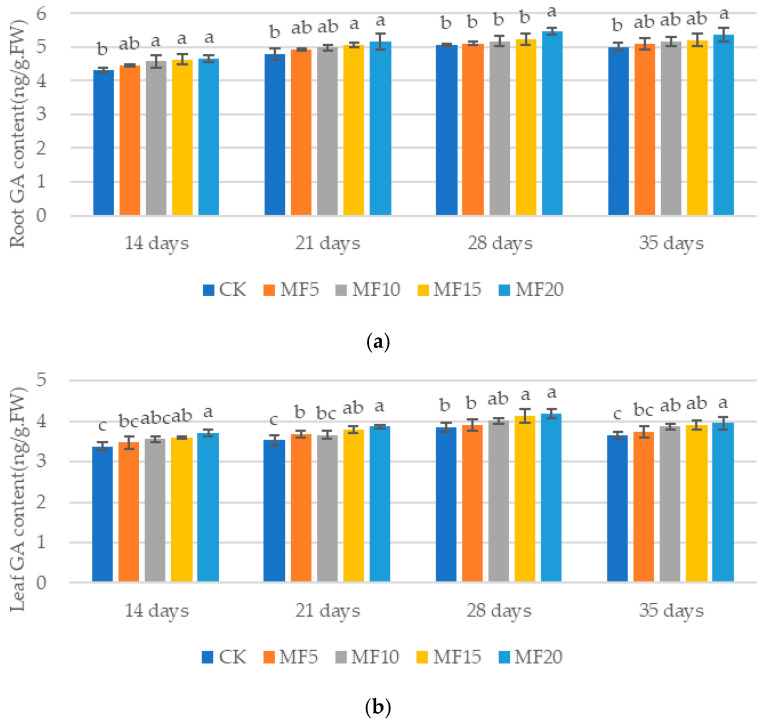
The impact of microbial fertilizer on the root (**a**) and leaf (**b**) GA contents of saline–alkali-stressed sugar beet seedlings. CK, MF5, MF10, MF15 and MF20 represent 0 kg, 5 kg, 10 kg, 15 kg and 20 kg microbial fertilizer applied to 300 kg saline–alkali soil, respectively; 14 days, 21 days, 28 days and 35 days correspond to the number of days after seedling emergence. Data are presented as the mean ± SD value, and lowercase letters represent significant differences among the means of the microbial fertilizer treatments (*p* ≤ 0.05).

As microbial fertilizer application increased across the five treatments, a gradual rise in GA content was observed in the sugar beet roots and leaves (Figure 9a,b). Compared with the CK, the GA content in the roots increased by 3.42–8.07%, 2.63–7.48%, 0.82–7.75% and 1.84–7.42% at 14 days, 21 days, 28 days and 35 days, respectively. The content in the leaves increased by 2.76–9.74%, 4.17–9.59%, 1.55–8.77% and 2.18–8.09%, respectively. The results of this study demonstrate that microbial fertilization facilitates the elevation of GA content in sugar beets in response to adversity stress.

**Figure 10:**
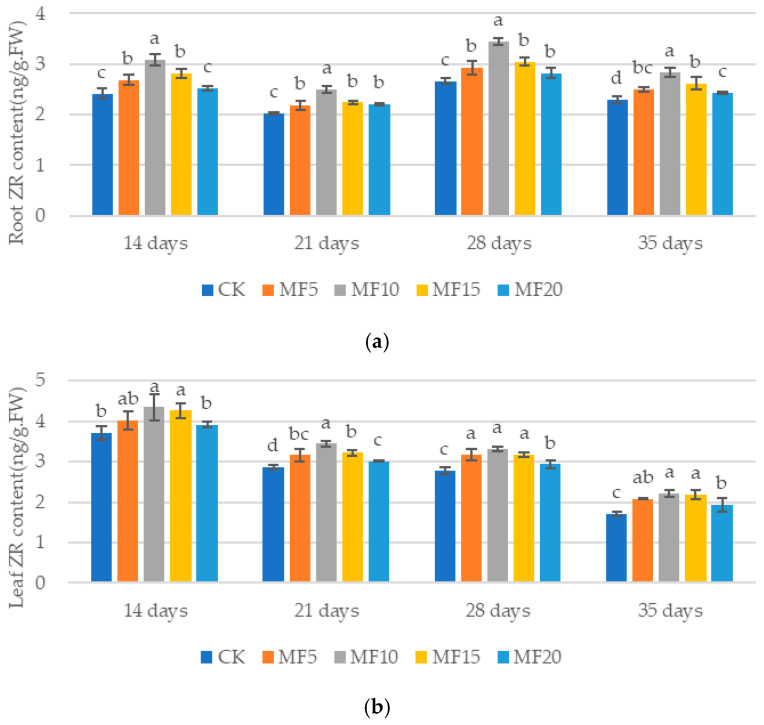
The impact of microbial fertilizer on the root (**a**) and leaf (**b**) ZR contents of saline–alkali-stressed sugar beet seedlings. CK, MF5, MF10, MF15 and MF20 represent 0 kg, 5 kg, 10 kg, 15 kg and 20 kg microbial fertilizer applied to 300 kg saline–alkali soil, respectively; 14 days, 21 days, 28 days and 35 days correspond to the number of days after seedling emergence. Data are presented as the mean ± SD value, and lowercase letters represent significant differences among the means of the microbial fertilizer treatments (*p* ≤ 0.05).

As microbial fertilizer application increased, the ZR contents in the roots and leaves of sugar beet exhibited an upward trend, followed by a subsequent decrease, across the five treatments (Figure 10a,b). Among these treatments, MF10 demonstrated the most significant increase compared to CK at 14 days, 21 days, 28 days and 35 days. The content in the MF10 roots increased by 28.07%, 23.17%, 29.35% and 23.58% and that in the MF10 leaves increased by 17.26%, 20.50%, 19.67% and 29.92%, respectively. This study suggests that ZR has the capacity to enhance plant resistance, thereby indicating that microbial fertilizer is advantageous for increasing the ZR content in sugar beet.

**Figure 11:**
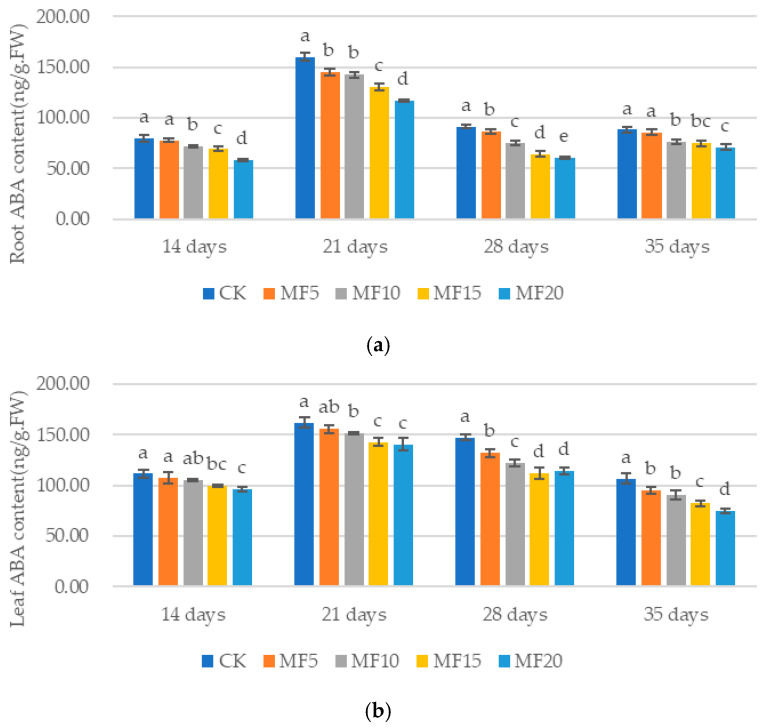
The impact of microbial fertilizer on the root (**a**) and leaf (**b**) ABA contents of saline–alkali-stressed sugar beet seedlings. CK, MF5, MF10, MF15 and MF20 represent 0 kg, 5 kg, 10 kg, 15 kg and 20 kg microbial fertilizer applied to 300 kg saline–alkali soil, respectively; 14 days, 21 days, 28 days and 35 days correspond to the number of days after seedling emergence. Data are presented as the mean ± SD value, and lowercase letters represent significant differences among the means of the microbial fertilizer treatments (*p* ≤ 0.05).

It was demonstrated that the application of microbial fertilizer increased the ABA contents in the sugar beet roots and leaves (Figure 11a,b). Compared with the CK, the concentration of ABA in the roots decreased by 2.12–27.16%, 9.48–26.97%, 5.38–33.39% and 3.20–19.46% at 14 days, 21 days, 28 days and 35 days, respectively. Correspondingly, the decreases in the leaves were determined to be 3.68–13.91%, 3.88–13.06%, 10.51–22.39% and 11.08–30.13%, respectively. It has been demonstrated that ABA, the hormone responsible for regulating plant responses to adversity, plays a pivotal role in the adaptation of plants to challenging conditions. This finding suggests that the microbial fertilizer effectively mitigates the effects of saline–alkali stress on sugar beet, as evidenced by the reduction in ABA content.

### 2.5. Transcriptome Sequencing and Alignment for Sugar Beet Seedlings

As demonstrated by the high-throughput sequencing results, a total of 164.92 Gb of base sequences were obtained from the roots and leaves of sugar beet seedlings. Contaminants were identified and eliminated, including those with an unknown base information content greater than 10%, as well as those with low-quality reads. It was determined that the Q20 of seedling roots and leaves was greater than 97.00%, the Q30 was greater than 91.93% and the GC content was above 41.52%, with a base error rate of 0.03%. These findings suggest that the entire library of this sample exhibited a high level of quality (Table 1).

### 2.6. Analysis of Differentially Expressed Genes

The DESeq2 analysis criterion was employed to study the gene expression of sugar beet roots and leaves under saline–alkali stress, with a cut-off of an adjusted *p*-value ≤ 0.05 and a log_2_ fold change ≥1.0. Different treatment samples were then compared two by two for differential gene analysis. As demonstrated in Table 2, a total of 388 differentially expressed genes were identified in the root system of the 14-day seedlings in the presence of microbial fertilizer, compared with the control group, which did not receive microbial fertilizer. This number includes 82 upregulated genes (21.13%) and 306 downregulated genes (78.87%).

### 2.7. Microbial Fertilization Regulates Important Genes in Saline–Alkali-Stressed Sugar Beet Seedlings

In contrast to the control group, the osmoregulation-related genes identified in the microbial fertilizer treatment roots of the 14-day seedlings included *ABCG25*, *At5g20260*, *SWEET4*, *ABCG32*, *At3g07620*, *BASS3*, *IRKI* and *SECA2*. Among these, *BASS3* expression was found to be downregulated, while the remaining genes exhibited an upregulation in expression. Antioxidant-related genes exhibited an elevated expression, predominantly *GSVIVT00023967001*. In the case of the signaling transduction genes, the most notable were *GA2OX2*, *D14* and *NCED2*. Among these, *D14* expression was found to be upregulated, while the remaining genes exhibited downregulation. In the roots of the 35-day seedlings, the predominantly expressed osmoregulation-related genes were *HAK17*, *UGT85A24* and *At5g26710*, all of which exhibited increased expression in the microbial fertilizer treatment relative to the control group; the predominantly expressed antioxidant-related genes were *APX3* and *APX1*, both of which demonstrated increased expression relative to the control group; and the signaling transduction genes predominantly expressed *ABP19A* and *ERF098*, both of which demonstrated increased expression relative to the control group (Table 3).

In the leaves of the 14-day seedlings, the differentially expressed antioxidant-related genes were predominantly *APX6* and *PER52*, both of which exhibited increased expression; the primary osmosis regulation-related gene was *AVT1A*, which also demonstrated increased expression; and the signaling transduction-related genes were mainly *ERF5* and *JOX4*, with *ERF5* exhibiting decreased expression and *JOX4* showing increased expression following microbial fertilizer treatment when compared to the control group. In the leaves of the 35-day seedlings, the predominantly expressed antioxidant-related gene was *PER44*; the main osmoregulation-related gene was *ABCG28*, which was upregulated; and those related to signaling transduction were *ERF017*, *CKX5*, *RALF*, *ERF061* and *ABP19A*, of which *ABP19A* showed increased expression and all others showed decreased expression following microbial fertilizer treatment when compared to the control group.

## 3. Discussion

### 3.1. Effect of Microbial Fertilizer on the Growth of Sugar Beet Seedlings Under Saline–Alkali Stress

Plant roots interact with soil microorganisms to form a unique microdomain, in which colonized microorganisms, such as bacteria and fungi, form a stable community structure through collaboration and competition. This is crucial for plant growth and development, disease resistance and stress tolerance [26]. For instance, the process of root colonization by inter-root-promoting bacteria, mycorrhizal fungi and symbiotic nitrogen-fixing bacteria has been demonstrated to directly enhance the capacity of plants to acquire nutrients from the soil [27], thereby facilitating the regulation of plant growth. Concurrently, microorganisms secrete pro-biotic substances or elicit an immune response, thereby inducing systemic resistance and ensuring optimal plant growth. It has been demonstrated that microorganisms have the capacity to secrete phytohormones, which, in turn, regulate primary root cell division and differentiation, thereby affecting root development and, consequently, regulating plant growth [28]. The present study posits that the utilization of microbial fertilizer has the capacity to enhance the retention rate of sugar beet seedlings. Furthermore, the employment of suitable microbial fertilizer has been demonstrated to facilitate an augmentation in root system size and leaf fresh weight. These observations are consistent with the findings of preceding studies.

### 3.2. Mechanisms of Microbial Fertilizers in Regulating the Roots of Sugar Beet Seedlings Under Saline–Alkali Stress

Osmotic substances are low-molecular-weight organic compounds, such as soluble sugars, proline and betaine, which help to regulate osmosis in plants by accumulating inside cells. This process is one way plants can maintain the expansion pressure of their cells, stabilize the active structure of enzymes in the cytoplasm and enhance their saline–alkali tolerance [29]. In this study, the research team sought to ascertain the effects of microbial fertilizer on osmotic homeostasis in sugar beet seedlings under saline–alkali stress. The study revealed that the microbial fertilizer regulated the differential expression of genes, such as *At5g20260*, *ABCG25* and *HAK17*, in the root system of sugar beet seedlings. The *At5g20260* gene encodes a protein that primarily catalyzes the synthesis of glycosidic bonds and transfers sugar residues from the donor substrate to the acceptor, thereby facilitating the synthesis of polysaccharides. The *ABCG25* gene encodes an energy-consuming transport protein that acts on a variety of molecules, including amino acids, inorganic ions and peptide-glycans. Finally, the *HAK17* gene encodes a potassium transporter. 

The application of an appropriate microbial fertilizer also had a significant effect on the accumulation of soluble sugars and free proline in the root system of sugar beet seedlings, where an increase in their accumulation assists in maintaining the water content in the cells, therefore allowing the plants to maintain osmotic homeostasis. Furthermore, the soluble protein content and the K^+^/Na^+^ ratio continued to improve with increasing microbial fertilizer concentration. It is well documented that Na^+^ is a toxic monovalent cation that causes damage to plant cells. K^+^ is a pivotal inorganic nutrient and osmotic regulator that is essential to the plant cell. The application of microbial fertilizer has been shown to promote saline–alkali stress tolerance in sugar beet seedlings, resulting in elevated K^+^ accumulation and Na^+^ reduction in the root system, consequently leading to an increased K^+^/Na^+^ ratio [30]. Betaine is an important organic osmolyte in plants that is involved in the resistance to saline–alkali stress, and the accumulation of betaine has been shown to have a positive effect on enzyme activity and membrane integrity [31]. In this study, the betaine content in the root system of sugar beet seedlings increased in the following sequence: MF5 > CK > MF10 > MF15 > MF20. This indicates that moderate microbial fertilizer facilitated the accumulation of betaine, which is advantageous to the cell membrane integrity required to maintained osmotic homeostasis.

In the context of saline–alkali stress, plants have been observed to accumulate substantial quantities of reactive oxygen species (ROS). A failure to remove these in a timely manner can result in a disruption of the dynamic equilibrium between the generation and removal of ROS, which, in turn, can lead to peroxidation and the degradation of membrane lipids. As a result, this can cause damage to membrane proteins and lipids, ultimately resulting in the destruction of the membrane structure due to the adverse effects of stress [32,33]. In this study, microbial fertilization was found to induce upregulation of the differentially expressed genes *APX1* and *APX3*, which encode ascorbate peroxidase, in the root system of sugar beet seedlings. The microbial fertilizer significantly promoted the levels of SOD, POD and CAT enzymes; increased the expression of antioxidant enzymes; reduced the malondialdehyde content in the root system; decreased the degree of membrane lipid peroxidation; enhanced the integrity of the membrane system; and facilitated the growth of sugar beet plants, all of which contribute to maintaining osmotic homeostasis.

Hormones have been demonstrated to play a pivotal regulatory role in the growth and development of plants. They are involved in the process of adaptive regulation of plants to saline–alkali environments, and it is imperative that phytohormones bind to hormone receptors in order to fulfill their physiological and biochemical roles. IAA is a notable plant growth hormone that can be synthesized by a variety of microorganisms [34]. In this study, microbial fertilization was found to significantly inhibit the expression of genes such as *GA2OX2*, *ABP19A* and *NCED2*. It is evident that *GA2OX2* plays a pivotal role in regulating the gibberellin balance in the root system of sugar beet seedlings. *ABP19A* is chiefly responsible for encoding a growth hormone-binding protein. *NCED2* is the key enzyme that limits the rate of abscisic acid biosynthesis. Appropriate microbial fertilizer application has been demonstrated to significantly increase IAA and ZR contents in the root system of sugar beets. Furthermore, GA content has been shown to exhibit a gradual increase, with enhanced signal transduction, thereby promoting the growth of sugar beets. ABA is a hormone that plays a regulatory role in the process of plant adaptation to adverse conditions. The present study demonstrated a decline in the amount of ABA with an increase in the application of microbial fertilizers. These findings suggests that microbial fertilizers can mitigate the adverse effects of saline–alkali stress on plants.

### 3.3. Mechanisms of Microbial Fertilizers in Regulating the Leaves of Sugar Beet Seedlings Under Saline–Alkali Stress

It has been demonstrated that in a saline stress environment, microorganisms have the capacity to alleviate osmotic stress and promote the adaptive response of plants to elevated saline concentrations. This process can be achieved through the production of osmotic substances or the modulation of the expression of genes related to osmotic substance regulation in plants [35]. Additionally, the accumulation of osmotic substances has been observed to alleviate osmotic stress. More specifically, the accumulation of osmotic substances within the soil microbiome has been demonstrated to enhance its capacity to adapt to the saline environment of the soil. This, in turn, functions to improve the inter-root environment under saline stress, thereby promoting nutrient uptake by the plant, enhancing the synthesis of osmotic substances and promoting plant growth.

In the context of saline–alkali stress conditions, the utilization of microbial fertilizer has been demonstrated to enhance the levels of soluble sugars within plant tissues. This, in turn, has been shown to result in an augmentation of plant salt tolerance [36]. In this study, the use of microbial fertilizer was found to induce the upregulation of *AVT1A* and *ABCG28*. *AVT1A*, in particular, is responsible for encoding amino acid transporters, a class of proteins that facilitate the transportation of amino acids across cell membranes. Meanwhile, *ABCG28* is responsible for encoding energy-consuming transport proteins for a wide range of molecules, including amino acids, inorganic ions and peptide-glycans. The application of appropriate microbial fertilizer resulted in a significant increase in the content of soluble sugars and free proline in the leaves of sugar beet seedlings. These substances are key osmoregulatory substances that reduce the osmotic potential of the cells and help maintain the normal physiological functions of plants [37]. The uptake of K^+^ increased significantly and the uptake of Na^+^ decreased significantly in plants inoculated with AMF (arbuscular mycorrhizal fungi) under salt stress [27]. Furthermore, many PGPRs (plant growth-promoting rhizobacteria) have been shown to increase K^+^ uptake and Na^+^ rejection in plants, thus increasing the K^+^/Na^+^ ratio [38]. In this study, the soluble protein content gradually increased with an increase in microbial fertilizer application, and the K^+^/Na^+^ ratio also continuously increased. The alterations in betaine content exhibited a high degree of congruence with the developmental patterns of the root system.

One established method of enhancing saline–alkali tolerance is to increase the antioxidant enzyme activity and enhance the level of antioxidant metabolism in plants. Inter-root microorganisms have been shown to activate antioxidant defense mechanisms in plants by upregulating the activities of certain antioxidant enzymes, thereby preventing membrane plasma damage caused by excess reactive oxygen species [31]. In this study, microbial fertilizer was found to upregulate the expression of *APX6* and *PER52*, both of which are involved in redox control. *APX6* encodes ascorbate peroxidase, an important enzyme component of the ascorbate (ASC)–glutathione (GSH) cycle in plant cells. It plays a significant role in scavenging cellular H_2_O_2_, and *PER52* encodes another important enzyme, a peroxidase. The application of microbial fertilizer demonstrated increased levels of SOD, POD and CAT enzymes in sugar beet seedlings, which was concomitant with a decline in malondialdehyde content and a decrease in membrane lipid peroxidation. These observations have been shown to result in improved plant cell viability and enhanced saline–alkali tolerance [39,40], thus indicating improved saline–alkali tolerance in sugar beet seedling with the application of microbial fertilizer.

Plant–microbe interactions have been demonstrated to alter microbial populations, thereby increasing plant tolerance to adverse environments. This alteration is achieved by affecting the gene expression of several signaling pathways, which represents a regulatory mechanism that is prevalent during saline–alkali stress [31]. It has been demonstrated that microorganisms have the capacity to promote plant growth through the production of growth hormones. In addition, they have been observed to enhance salt tolerance by regulating growth hormone levels and inducing a range of stress responses in plants [41,42]. In this study, microbial fertilizers were observed to downregulate the expression of *ERF5*, *CKX5* and *ERF061* and upregulate the expression of *JOX4* and *ABP19A*. *ERF5* and *ERF061* encode ethylene response factors, while *CKX5* encodes a cytochrome P450, which is involved in the degradation of jasmone acid (JA). The main function of this enzyme is to break the side chain of JA and inactivate it. *JOX4* encodes a protein whose main function is to convert JA to 12-OH-JA, a process that is essential in the plant defense system. Finally, *ABP19A* encodes a growth-hormone-binding protein. Appropriate microbial application increased the IAA and ZR contents of sugar beet leaves, while the GA content demonstrated a gradual increasing trend and the ABA content decreased, indicating that the saline–alkali tolerance of microbial-fertilizer-regulated sugar beet seedlings was primarily attributed to the increase in the contents of endogenous IAA, ZR and GA, as well as the decrease in the content of ABA. This suggests that the microbial fertilizer induced a variety of hormones to work together to improve the saline–alkali tolerance of the plant

## 4. Materials and Methods

### 4.1. Plant Cultivation and Treatments

The preliminary potting test was conducted by extracting soil from the 0 to 20 cm tillage layer of the saline–alkali land at the Bayannur Academy of Agricultural & Animal Sciences. The base nutrients were ascertained to be 17.1 g/kg of organic matter, 60.4 mg/kg of alkaline dissolved nitrogen, 27.2 mg/kg of effective phosphorus, 165.3 mg/kg of quick-acting potassium and 0.34% of saline–alkali compounds. The pH was 8.8, and the soil was sifted with a sieve hole diameter of ≤1 cm prior to potting. The sugar beet variety under study is designated KWS2314. A total of 12 pots were utilized for each treatment; the soil and fertilizer were thoroughly mixed prior to filling and each pot was filled with 3 kg. A total of 20 seeds were sown for cultivation under the following conditions: temperature 25 °C/20 °C day/night, light intensity 450 µmol/m s, relative humidity 70% and photoperiod 13 h/11 h light/dark. Sugar beets are cultivated using de-ionized water.

The results of the preliminary test were used to determine the amount of microbial fertilizer to be applied. The following design was employed for the microbial fertilizer application per 300 kg of saline–alkali soil: 0 kg (CK), 5 kg (MF5), 10 kg (MF10), 15 kg (MF15) and 20 kg (MF20). The tissue samples were taken from the sugar beet seedlings, roots and leaves. Tissue samples of the roots and leaves were collected at 14 days, 21 days, 28 days and 35 days after seedling emergence and stored at −80 °C for subsequent analysis. The microbial bacterial fertilizer was a saline soil conditioner provided by the China National Research and Promotion Center of Yield Enhancing Bacteria Technology and the Pilot Base of Agricultural Biological Agents of China Agricultural University. The main components of this fertilizer are Bacillus subtilis, dephosphorylated bacteria and Potassium-solubilizing bacteria, and the effective living bacterial count is 2.0 billion/g.

### 4.2. Sugar Beet Seeding Retention and Fresh Weight

This study investigated sugar beet seedling retention at 35 days after seedling emergence. The experiment involved washing the plants and separately weighing the fresh weights of the roots and leaves.

### 4.3. Determination of Osmoregulatory Substances in Sugar Beet

The quantification of soluble sugar was conducted as follows: 20 mg of dry powder from each beet sample was weighed, 500 µL of distilled water was added and the mixture was boiled for 10 min to extract the sugar. Following this extraction, the mixture was subjected to centrifugation at 12,000 rpm for 10 min at room temperature to remove the precipitate. This process was repeated three times to obtain three technical replicates. The resultant clear upper layer was designated as the crude extract and the final sample solution was diluted 100 fold. The quantity of soluble sugars was then determined through enthrone colorimetry. The proline content was determined by referring to the study in [43], and the related sample pretreatment and assay methods were optimized and improved. The determination of betaine content was achieved through the utilization of [44] as a reference, with the pretreatment and detection methods for the related sample being optimized and improved. The determination of soluble protein content was achieved through the weighing of 5 mg of beet powder for each sample, which was subsequently added to 0.15 mL of 1 M KH_2_PO_4_ + K_2_HPO_4_ extraction buffer. The extraction process was conducted under ultrasonic conditions at 4 °C for a duration of 30 min. Thereafter, the mixture was subjected to centrifugation at 12,000 rpm for a period of 20 min, with the objective of removing the precipitate. The resultant clear layer, which corresponded to the crude extract of total protein, was then subjected to quantification via the Bradford method. The determination of malondialdehyde content was achieved through the following procedure: Initially, a quantity of sample powder was transferred into a centrifuge tube. Then, a small hole was made in the lid of the tube using a needle. The sample was then mixed thoroughly using a vortex mixer. Thereafter, the tube was placed in a water bath at 95 °C for a period of 40 min to remove. Following incubation, each sample was removed from the water bath and cooled under running water. Subsequently, the samples were centrifuged for 10 min at 3500–4000 rpm. The upper layer of the mixture was designated as the MDA extract, which was subsequently measured using the TBA method. The contents of K^+^ and Na^+^ were determined by employing HNO_3_ digestion, with the dried beet powder samples undergoing a pre-treatment using an ICP-OES method. The contents of various metal elements were subsequently determined by means of an inductively coupled plasma emission spectrometer (iCAP 6300 ICP-OES Spectrometer, Thermo Fisher, Waltham, MA, USA).

### 4.4. Determination of Antioxidant Enzyme Levels in Sugar Beet

The kit employs a double-antibody one-step sandwich enzyme-linked immunosorbent assay (ELISA) technique. The configuration of the standard gradient was as follows: 320, 160, 80, 40, 20 and 10 U/mL. For each sample, 50 µL of sample extract was applied to the ELISA plate. Furthermore, in instances where the value of certain samples exceeded the maximum standard concentration, the sample was further diluted such that the result fell within the range of the standard curve. The absorbances (OD values) were measured at a wavelength of 450 nm using an enzyme marker. The contents of plant SOD, POD and CAT enzymes in the final sample were then calculated based on the standard curve.

### 4.5. Measurement of IAA, GA, ZR and ABA

The IAA, GA, ZR and ABA contents were measured using enzyme-linked immunosorbent assay (ELISA) kits provided by China Agricultural University. Fresh leaves (1 g) were homogenized in liquid nitrogen, and hormones were extracted using 2 mL of extraction buffer (80% methanol with 1 mM butylated hydroxytoluene). The mixture was stored at 4 °C for 4 h and centrifuged at 3500 rpm for 8 min at 4 °C. The resulting supernatant was treated with a C18 solid-phase extraction column and the analytes were eluted from the column using 2.5 mL of 100% (*v*/*v*) methanol. The extract was vacuum-evaporated to remove the methanol, and the evaporated residue was dissolved with 2 mL of PBS buffer containing Tween 20 (0.1% [*v*/*v*]) and 1 g/L gelatin. ELISA measurement was performed on a 96-well microtitration plate provided by the manufacturer. Each well contained either 50 µL of an extract or standard and 50 µL of 5 µg/mL antibodies against IAA, GA, ZR or ABA. Each plate was incubated for 1 h at 37 °C and then washed four times with washing buffer (PBS buffer containing 0.1% Tween 20). A total of 100 µL of 1.25 µg/mL IgG-horseradish peroxidase substrate was added to each well and the reaction was incubated for 30 min at 30 °C. The microtitration plate was washed four times as above, and then 100 µL color-appearing solution containing 1 mg/mL O-phenylenediamine and 0.008% (*v*/*v*) H_2_O_2_ was added to each well. The reaction was stopped by adding 50 µL of 2M H_2_SO_4_. The OD values at 490 nm of each sample were determined using an ELISA reader. The IAA, GA, ZR and ABA contents in the final samples were then calculated based on the standard curve.

### 4.6. cDNA Library Preparation and RNA-Seq

The root and leaf transcriptome sequencing was performed on days 14 and 35 of the CK and MF10 treatments. This was based on the growth and physiological and biochemical parameters of sugar beet. The Illumina sequencing platform is the basis of a widely utilized method of transcriptome sequencing and was employed to study all mRNAs transcribed from sugar beet tissues at a specific time point. RNA-seq has become a major method for transcriptome research due to its advantages of high throughput, high sensitivity and broad applications. The RNA-seq technology process comprises the following two primary components: construction of libraries and bioinformatic analysis. The RNA was extracted from the tissues using standard extraction methods, followed by rigorous quality control of the RNA samples. This was mainly carried out using an Agilent 2100 bioanalyzer to accurately detect RNA integrity. Following the successful completion of the library inspection, the various libraries were amalgamated according to their effective concentration and the target downstream data volume for Illumina sequencing.

### 4.7. Identification of Differentially Expressed Genes

The present study investigated the gene expression of sugar beet roots and leaves under saline–alkali stress. Differential gene screening was performed by pairwise comparison of each microbial treatment and tissue type and its corresponding control. The DESeq2 platform was used to conduct the analysis with screening criteria of an adjusted *p*-value ≤ 0.05 and a log_2_ fold change ≥1.0.

### 4.8. Statistical Analysis

The data obtained are expressed as the mean and standard error, and all of the experiments were repeated three times. Analysis of variance (ANOVA) between the physiological parameters of plants in the control and saline–alkali treatments was performed using SAS 9.0.

## 5. Conclusions

In this study, the response mechanism of saline–alkali-stressed sugar beet seedlings to microbial fertilization was analyzed using physiology and transcriptomics. The application of microbial fertilizer is shown to have a significant impact on the expression of genes associated with osmoregulation, antioxidant enzymes and phytohormones in sugar beet seedlings under saline–alkali stress. The use of these fertilizers is demonstrated to result in a notable increase in the levels of soluble sugars, soluble proteins and free proline in the roots and leaves of sugar beet seedlings. This study demonstrates a reduction in MDA content, an increase in K^+^/Na^+^ ratio and an enhancement of the activities of antioxidant enzymes SOD, POD and CAT. The results also indicate the induction of the hormones IAA, GA and ZR. Furthermore, this study reveals that the contents of IAA, GA and ZR increased, while the content of ABA decreased. As a result, this study found a significant increase in seedling retention and fresh weight of sugar beet seedlings.

## Figures and Tables

**Table 1:** Transcriptome data of roots and leaves of sugar beet seedlings in the control group and following treatment with microbial fertilizer.

Sample	Raw Reads	Raw Bases	Clean Reads	Clean Bases	Error_ Rate	Q20	Q30	GC (%)
CKR141	47753286	7.16 G	46780204	7.02 G	0.03	97.22	92.43	42.94
CKR142	47589848	7.14 G	46715122	7.01 G	0.03	97.31	92.74	43.01
CKR143	41420298	6.21 G	40502964	6.08 G	0.03	97.15	92.30	42.96
MFR141	45841150	6.88 G	44684554	6.70 G	0.03	97.35	92.77	42.93
MFR142	42508768	6.38 G	41146434	6.17 G	0.03	97.31	92.61	42.74
MFR143	47536994	7.13 G	46153018	6.92 G	0.03	97.11	92.18	42.88
CKR351	46944566	7.04 G	45633760	6.85 G	0.03	97.24	92.47	42.38
CKR352	49176016	7.38 G	48067686	7.21 G	0.03	97.30	92.54	41.97
CKR353	42532576	6.38 G	41035740	6.16 G	0.03	97.26	92.53	41.52
MFR351	41906002	6.29 G	40858146	6.13 G	0.03	97.02	91.93	42.01
MFR352	46465650	6.97 G	45025034	6.75 G	0.03	97.14	92.21	42.09
MFR353	43744572	6.56 G	43043216	6.46 G	0.03	97.39	92.85	42.78
CKL141	47319126	7.10 G	46530012	6.98 G	0.03	97.38	92.69	43.61
CKL142	44370914	6.66 G	42726458	6.41 G	0.03	97.45	92.93	43.38
CKL143	45810000	6.87 G	44462518	6.67 G	0.03	97.61	93.31	43.70
MFL141	46487394	6.97 G	45105062	6.77 G	0.03	97.59	93.19	43.68
MFL142	48043430	7.21 G	46680844	7.00 G	0.03	97.04	92.14	43.68
MFL143	51087790	7.66 G	49202892	7.38 G	0.03	97.33	92.64	43.12
CKL351	46409334	6.96 G	45000052	6.75 G	0.03	97.48	92.95	42.79
CKL352	45967052	6.90 G	44912846	6.74 G	0.03	97.00	92.04	42.61
CKL353	46424230	6.96 G	45259438	6.79 G	0.03	97.45	92.89	42.69
MFL351	41423172	6.21 G	40703336	6.11 G	0.03	97.25	92.42	42.42
MFL352	45310110	6.80 G	43811570	6.57 G	0.03	97.26	92.42	42.52
MFL353	47306530	7.10 G	46569176	6.99 G	0.03	97.19	92.19	42.62

CK and MF indicate 0 kg and 10 kg microbial fertilizer applied to 300 kg saline–alkali soil; R and L indicate root and leaf; 14 and 35 are the number of days after seedling emergence; and 1, 2 and 3 indicate the different replicates.

**Table 2:** Statistics of the differentially expressed genes in sugar beet seedlings following treatment with microbial fertilizer.

Comparison	All Differentially Expressed Genes	Upregulated Genes	Downregulated Genes
MFR14 vs. CKR14	388	82	306
MFR35 vs. CKR35	171	107	64
MFL14 vs. CKL14	102	79	23
MFL35 vs. CKL35	418	175	243

**Table 3:** Important differentially expressed genes in sugar beet following treatment with microbial fertilizer.

Treatment	Gene Classification	Gene ID	Gene Name	Gene Description (Swissprot Database)	Regulation Pattern
MFR14 vs. CKR14	Osmoregulation	IMABv01g023760	*ABCG25*	ABC transporter G family member 25	Up
		IMABv02g032324	*At5g20260*	Probable glycosyltransferase	Up
		novel.664	*SWEET4*	Bidirectional sugar transporter	Up
		IMABv08g029340	*ABCG32*	ABC transporter G family member 32	Up
		IMABv07g015513	*At3g07620*	Probable glycosyltransferase	Up
		IMABv05g000506	*BASS3*	Probable sodium/metabolite cotransporter	Down
		IMABv04g008626	*IRKI*	IRK-interacting protein	Up
		novel.3487	*SECA2*	Protein translocase subunit	Up
	Antioxidant	IMABv09g020441	*GSVIVT00023967001*	Peroxidase 4	Down
	Signal transduction	IMABv05g000249	*GA2OX2*	Gibberellin 2-beta-dioxygenase	Down
		IMABv05g000235	*GA2OX2*	Gibberellin 2-beta-dioxygenase 2	Down
		IMABv08g029383	*D14*	Strigolactone esterase	Up
		IMABv04g006193	*NCED2*	9-cis-Epoxycarotenoid dioxygenase	Down
MFR35 vs. CKR35	Osmoregulation	IMABv06g019546	*HAK17*	Probable potassium transporter 17	Up
		IMABv03g009139	*UGT85A24*	7-Deoxyloganetin glucosyltransferase	Up
		IMABv05g000475	*At5g26710*	Glutamate-tRNA ligase cytoplasmic	Up
	Antioxidant	IMABv06g020044	*APX3*	L-ascorbate peroxidase 3	Up
		novel.2533	*APX3*	L-ascorbate peroxidase 3	Up
		IMABv09g021299	*APX1*	L-ascorbate peroxidase 1	Up
	Signal transduction	IMABv08g028764	*ABP19A*	Auxin-binding protein	Up
		novel.1826	*ERF098*	Ethylene-responsive transcription factor	Up
MFL14 vs. CKL14	Osmoregulation	IMABv03g00921	AVT1A	Amino acid transporter	Up
	Antioxidant	IMABv02g030636	*APX6*	Putative L-ascorbate peroxidase 6	Up
		IMABv02g031865	*PER52*	Peroxidase 52	Up
	Signal transduction	IMABv05g003391	*ERF5*	Ethylene-responsive transcription factor 5	Down
		novel.3195	*JOX4*	Jasmonate-induced oxygenase 4	Up
MFL35 vs. CKL35	Osmoregulation	IMABv07g0148989	*ABCG28*	ABC transporter G family member 28	Up
	Antioxidant	IMABv05g002263	*PER44*	Peroxidase 44	Down
	Signal transduction	IMABv08g028833	*ERF017*	Ethylene-responsive transcription factor	Down
		IMABv01g024418	*CKX5*	Cytokinin dehydrogenase 5	Down
		IMABv03g008822	*RALF*	Rapid alkalinization factor	Down
		IMABv05g004284	*ERF061*	Ethylene-responsive transcription factor	Down
		IMABv08g028764	*ABP19A*	Auxin-binding protein	Up

## Data Availability

RNA-seq sequencing data have been deposited in the SRA, under accession number: PRJNA1281816.

## Abbreviations

The following abbreviations are used in this manuscript:

MDA	Malonaldehyde
SOD	Superoxide dismutase
POD	Peroxidase
CAT	Catalase
IAA	Indole acetic acid
GA	Gibberellic acid
ZR	Zeatin riboside
ABA	Abscisic acid
FW	Fresh weight
ROS	Reactive oxygen species
AMF	Arbuscular mycorrhizal fungi
PGPRs	Plant growth-promoting rhizobacteria
JA	Jasmone acid
